# 4D dose simulation in volumetric arc therapy: Accuracy and affecting parameters

**DOI:** 10.1371/journal.pone.0172810

**Published:** 2017-02-23

**Authors:** Thilo Sothmann, Tobias Gauer, René Werner

**Affiliations:** 1 Department of Computational Neuroscience, University Medical Center Hamburg-Eppendorf, Hamburg, Hamburg, Germany; 2 Department of Radiotherapy and Radio-Oncology, University Medical Center Hamburg-Eppendorf, Hamburg, Hamburg, Germany; North Shore Long Island Jewish Health System, UNITED STATES

## Abstract

Radiotherapy of lung and liver lesions has changed from normofractioned 3D-CRT to stereotactic treatment in a single or few fractions, often employing volumetric arc therapy (VMAT)-based techniques. Potential unintended interference of respiratory target motion and dynamically changing beam parameters during VMAT dose delivery motivates establishing 4D quality assurance (4D QA) procedures to assess appropriateness of generated VMAT treatment plans when taking into account patient-specific motion characteristics. Current approaches are motion phantom-based 4D QA and image-based 4D VMAT dose simulation. Whereas phantom-based 4D QA is usually restricted to a small number of measurements, the computational approaches allow simulating many motion scenarios. However, 4D VMAT dose simulation depends on various input parameters, influencing estimated doses along with mitigating simulation reliability. Thus, aiming at routine use of simulation-based 4D VMAT QA, the impact of such parameters as well as the overall accuracy of the 4D VMAT dose simulation has to be studied in detail–which is the topic of the present work. In detail, we introduce the principles of 4D VMAT dose simulation, identify influencing parameters and assess their impact on 4D dose simulation accuracy by comparison of simulated motion-affected dose distributions to corresponding dosimetric motion phantom measurements. Exploiting an ITV-based treatment planning approach, VMAT treatment plans were generated for a motion phantom and different motion scenarios (sinusoidal motion of different period/direction; regular/irregular motion). 4D VMAT dose simulation results and dose measurements were compared by local 3% / 3 mm *γ*-evaluation, with the measured dose distributions serving as ground truth. Overall *γ*-passing rates of simulations and dynamic measurements ranged from 97% to 100% (mean across all motion scenarios: 98% ± 1%); corresponding values for comparison of different day repeat measurements were between 98% and 100%. Parameters of major influence on 4D VMAT dose simulation accuracy were the degree of temporal discretization of the dose delivery process (the higher, the better) and correct alignment of the assumed breathing phases at the beginning of the dose measurements and simulations. Given the high *γ*-passing rates between simulated motion-affected doses and dynamic measurements, we consider the simulations to provide a reliable basis for assessment of VMAT motion effects that–in the sense of 4D QA of VMAT treatment plans–allows to verify target coverage in hypofractioned VMAT-based radiotherapy of moving targets. Remaining differences between measurements and simulations motivate, however, further detailed studies.

## Introduction

Radiation therapy of lung and liver lesions has fundamentally changed from conventional 3D conformal radiation therapy (3D-CRT) to hypofractioned and even ablative-type treatment schemes such as stereotactic body radiation therapy (SBRT) or stereotactic ablative radiotherapy (SABR) [1, 2]. Treatment plans are often delivered by intensity modulated radiation therapy (IMRT)-type techniques like volumetric modulated arc therapy (VMAT) [3]. Lung and liver lesions are, however, subject to respiratory motion with well-studied motion amplitudes of up to several centimetres [4, 5]. For IMRT-type dose delivery, the target motion may lead to a risk of so-called interplay effects, i. e. the unintended interference of target motion and dynamically changing beam parameters such as gantry position and MLC segments’ shape [6]. Some years ago, this has been an oft-reported reason to avoid IMRT techniques for lung and liver treatment even for conventional fractionation schemes [7]–although planning studies highlighted superior dose distribution characteristics compared to 3D-CRT [8, 9] and related interplay effects were shown to average out over the course of treatment [10].

Nowadays, lung and liver patients *are* treated in a few or even a single fraction [11], with high target doses delivered by VMAT techniques (sometimes even by means of only a single arc [12]) and employing treatment units with high dose rates, e. g. using flattening filter free beams [13, 14]. Thus, the risk associated to single fraction interplay effects appears to be increased. This, on the one hand, highlights the importance of studies on interplay effects in the context of VMAT and hypofractionation [6, 14–21]. On the other hand and from a clinical perspective, it also motivates establishing 4D quality assurances (4D QA) to assess the potential risk of motion and interplay effects for generated treatment plans when accounting for patient-specific breathing patterns before dose delivery. Related studies, including the aforementioned references, can be divided into computational 4D dose simulation-based and motion phantom, i. e. measurement-based approaches. A drawback of motion phantom-based 4D QA is that it is restricted to a small(er) number of measurements and variations of the patient-specific motion patterns. In contrast, computational approaches allow simulating almost every conceivable scenario and have the potential to provide a more comprehensive picture of motion effects and their impact on, e. g., target coverage. They, however, require instantiation of various input parameters that influence estimated dose distributions–and which therefore mitigate reliability of the simulation results.

Being placed in the in the context of the discussion about appropriate VMAT 4D QA, the present study aims to analyze the impact of 4D VMAT dose simulation parameters on the simulation accuracy, to identify the most influencing factors, and finally to assess the overall appropriateness of 4D VMAT dose simulation to assess motion-induced dose alterations for VMAT-based SBRT treatment when compared to motion phantom-based measurements. To do so, we built on a computational 4D dose simulation scheme originally introduced for the analysis of interplay effects in step-and-shoot IMRT [7], extended it to VMAT dose delivery, and studied different physiological as well as technical parameters that likely affect 4D VMAT dose simulation accuracy.

## Materials and methods

This section is structured as follows: First, the concept of VMAT dose delivery is introduced, the fundamentals of the applied 4D dose simulation scheme are described and parameters are identified that potentially influence 4D VMAT dose simulation accuracy. Finally, the study design, i. e. strategies to address these parameters, and the performed experiments are detailed.

### Principles of and uncertainties in 4D VMAT dose simulation

**Fig 1:**
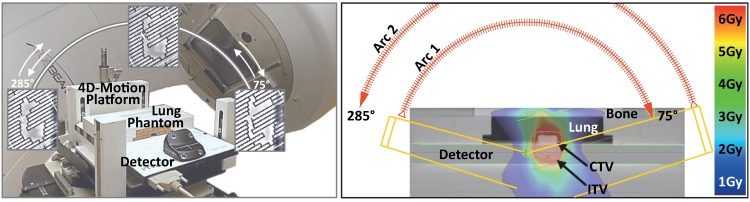
Experimental setup. Left: Measurement setup: 4D motion platform with detector array and lung phantom, consisting of bone, lung and tissue equivalent materials. Right: Average CT of setup, planned VMAT dose distribution, and target structures/organs at risk.

Volumetric arc therapy exploits dose rate and gantry speed variation as well as leaf modulation to maximize benefits of classic (e. g. static field) IMRT techniques [22]. Briefly speaking, the dose is delivered during continuous gantry rotation around the patient, usually covering a wide range of gantry angles and often using a (small) number of so-called arcs as indicated in Fig 1.

Due to leaf modulation, each gantry angle is further associated with a multileaf collimator (MLC)-formed treatment field of specific shape and dose; cf. Fig 1 (left).

Computer-based simulation of VMAT dose delivery requires discretization of the continuous process. Let $\dot{D}\;:\;\Omega \subset R^{3}\times T\subset R\to R_{+}$ be the time-dependent dose rate of the dose delivery process and $D\;:\Omega \to R_{+}$ the resulting dose distribution; then, the discretization mathematically translates into

$$D\left(x\right)=\int_{T}\dot{D}\left(x,t\right)\;dt\approx \sum_{t\in \tilde{T}}\dot{D}\left(x,t\right)\Delta t=\sum_{t\in \tilde{T}}D_{t}\left(x\right)$$ (1)

with T=(0,T] being the period of dose delivery and $\tilde{T}=\left\{\Delta t,2\Delta t,\ldots ,T\right\}$ a sampled version of T. In other words: $D_{t}(x)≔\dot{D}(x,t)\Delta t$ represents an approximation of the dose delivered during the interval (t-Δt,t]⊂T, and the sum of all *D*_*t*_(*x*)–the *accumulated* or simulated (4D) dose [7]–approximates the entire dose *D* to the voxel at spatial position *x* ∈ Ω.

Focusing on single arc VMAT dose delivery, the time variable *t* can be replaced by the gantry rotation angle *α*, and Eq (1) reads

$$D\left(x\right)\approx \sum_{\alpha \in \tilde{A}}\dot{D}\left(x,\alpha \right)\Delta \alpha =\sum_{\alpha \in \tilde{A}}D_{\alpha }\left(x\right)$$ (2)

with $\tilde{A}\subset A$ being a discretized version of the gantry angle range A of the considered arc. As before, $D_{\alpha }(x)≔\dot{D}(x,\alpha )\Delta \alpha$ is an approximation of the dose delivered to *x* during the gantry interval (*α* − Δ*α*, *α*]. Thus, for Δ*α* → 0, the distributions *D*_*α*_ can be interpreted as the dose delivered for the individual MLC-formed fields illustrated in Fig 1 (left).

So far, Eqs (1) and (2) represent a discretization of single arc VMAT dose delivery to a *static* geometry. Including (here: breathing-induced) motion means to additionally account for a time dependence of the spatial voxel position during dose delivery. This leads to a common modification of Eq (2) [23]:

$$D_{\text{dyn}}\left(x\right)\approx \sum_{\alpha \in \tilde{A}}\sum_{t\in {\tilde{T}}_{\alpha }}D_{\alpha }\left(\varphi_{t}\left(x\right)\right)=\sum_{\alpha \in \tilde{A}}\sum_{t\in {\tilde{T}}_{\alpha }}\left(D_{\alpha }\circ \varphi_{t}\right)\left(x\right).$$ (3)

Here, ${\tilde{T}}_{\alpha }$ represents a sampled version of the time interval corresponding to dose delivery for the gantry angle interval (*α* − Δ*α*, *α*] and *φ*_*t*_(*x*) ∈ Ω the position of the voxel originally positioned at *x* at time point $t\in {\tilde{T}}_{\alpha }$. The other way around, $\varphi_{t}\;:\Omega \to R^{3}$ can also be read as a transformation that maps the dose delivered to the correspondingly moved geometry *φ*_*t*_(Ω) to the original (= reference) coordinate space and geometry Ω, with the latter being usually defined by a pre-selected phase of a planning 4D CT or a derived image like a temporal average CT. Consequently, the resulting dose distribution $D_{\text{dyn}}\;:\Omega \to R^{3}$ represents an estimation of the dose delivered during the considered VMAT arc and accounting for the geometry (= patient, phantom) motion represented by the set of transformations *φ*_*t*_ representing all $t\in {\tilde{T}}_{\alpha }$ and $\alpha \in \tilde{A}$. Note that the nested summation in Eq (3) is only necessary if the temporal resolution of the target motion information is higher than the resolution of the dose delivery process; otherwise, the 4D dose simulation can be formulated by a single summation.

Extending single arc 4D dose simulation, i. e. Eq (3), to an entire treatment course would finally lead to an extended nested summation,

$$D_{\text{dyn}}^{\text{total}}\;\left(x\right)=\underset{\text{total treatment dose}}{\underset{︸}{\overset{n_{\text{fx}}}{\underset{i=1}{\sum }}\;\underset{\text{treatment fraction dose}}{\underset{︸}{\overset{n_{\text{arc}}}{\underset{j=1}{\;\sum }}\;\underset{\text{single arc dose}}{\underset{︸}{\underset{\alpha \in {\tilde{A}}_{j}}{\sum }\;\underset{\text{angle interval dose}}{\underset{︸}{\underset{t\in {\tilde{T}}_{\alpha }}{\sum }\left(D_{i,j,\alpha }\circ \varphi_{i,j,t}\right)\left(x\right)}}}}}}}}.$$ (4)

Assuming more or less periodic motion patterns, the fractionation effect (outermost summation) on the delivered dose distribution can be simulated by repeating treatment fraction dose computation with randomly varied breathing phases associated to the beginning of the dose delivery of the individual fractions (i. e. randomly shifting the voxel trajectories in time) and summing up the dose distributions [7]. This averages out single fraction motion and interplay effects, depending on the number of fractions *n*_fx_. Principle and dosimetric consequences have already been well-explained for classic IMRT techniques [10], and related conclusions can be transferred to VMAT. Similar observations can also be made for the summation over different arcs [21].

As our study aims to analyze factors that systematically (i. e. in a deterministic way) influence 4D dose simulation accuracy, we refrain from considering the randomness and averaging effects induced by fractionation and multi-arc dose delivery. Instead, we focus on single arc 4D dose simulation as given by the innermost summation and Eq (3), respectively.

Further neglecting implementation details such as the exact type of dose interpolation in Eq (3) and potential effects of, e. g., ignoring mass effects by not applying energy transformation models (previously proven to be small for small voxel sizes [23]), the following parameters are considered as potentially most affecting 4D VMAT dose simulation accuracy and are studied:

P1 *Accuracy of target structure and organs at risk motion representation* Correct representation of target structure and organs at risk motion, i. e. the estimation of the transformations *φ*_*i*,*j*,*α*_, is a prerequisite for accurate 4D dose simulation and assessment of motion/interplay effects. In real patient-scenarios, the *φ*_*i*,*j*,*α*_ are usually computed by non-linear registration in planning 4D CT data–with the problem of being confined by a limited temporal resolution of the data and uncertainties associated to the applied registration approach; see Ref. [24, 25] for recent overviews on non-linear registration accuracy in 4D CT image sequences. Phantom studies, however, usually allow applying known motion patterns, which reduces these uncertainties to a minimum. Imperfect synchronization of the phantom breathing phase at measurement beginning and the assumed starting phase used for simulation purposes remains nevertheless as source of error especially for comparison of measurements and simulations.
P2 *Degree of temporal discretization of technical dose delivery process* Discretization of the continuous VMAT dose delivery process, i. e. Eqs (2) and (3), means to ignore potential interplay effects that are due to gantry or MLC leaf movements during the considered intervals of size Δ*t* and Δ*α*. Thus, interplay effects on a times scale of Δ*t* (Δ*α*) will not be represented by the simulated 4D dose.
P3 *Accuracy of static dose calculation* Like for any RT treatment planning, 4D dose simulation accuracy also depends on the dose calculation algorithm and dose grid size (has to be small for interpolation-based 4D dose simulation schemes) applied for computation of the individual *D*_*i*,*j*,*α*_. In addition and especially for lung SBRT, the choice of appropriate CT images and density distributions considered for *D*_*i*,*j*,*α*_ computation further remains an open issue and a potentially influencing parameter when comparing 4D dose simulation results to measurements.

### Study design and experiments

Following the previous section, our study aimed at analyzing the influence of the above-mentioned aspects on single fraction single arc 4D dose simulation accuracy by comparison of motion phantom dose measurements and corresponding simulated dose distributions; the measurements were considered as ground truth to be resembled by the simulations as closely as possible. The motion phantom setup has already been shown in Fig 1 and is–together with the applied dose measurement equipment, treatment planning aspects, motion scenarios, the performed experiments and our evaluation strategy–detailed in the following.

#### Motion phantom and dose measurement setup

The motion phantom consisted of a lung phantom add-on compiled by solid-water, lung, tumor and bone inserts, and the Octavius 1000 SRS detector array (PTW Freiburg, Germany). The detector provided high spatial resolution dose measurements by means of 977 liquid filled ionization chambers, distributed over an area of 11×11 cm, with a 2.5 mm chamber spacing in the inner 5.5×5.5 cm and 5 mm spacing in the outer detector area [26]. The add-on was mounted on a computer-controlled motion platform with three spatial degrees of freedom (Euromechanics, Germany). According to Ref. [27], the phantom-based regular breathing pattern simulation accuracy is higher than 0.5 mm.

#### Motion scenarios and treatment planning

**Fig 2:**
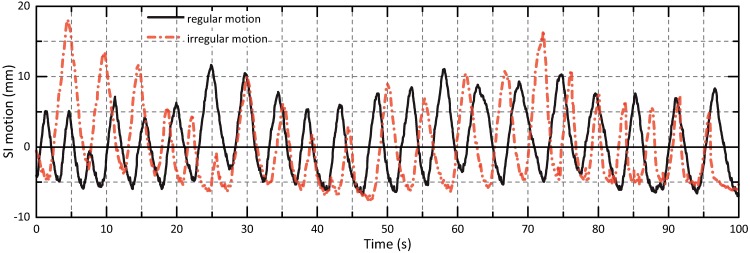
Patient motion scenarios. SI motion amplitudes of applied regular and irregular tumor trajectories.

The characteristics of the motion patterns programmed to the phantom are listed in Table 1. Five sinusoidal curves with varying motion period and directions as well as two real-patient tumor motion paths extracted from CyberKnife lung SBRT treatment logfiles were considered. The (ir)regularity of the real-patient curves differed significantly (Fig 2); the two scenarios are subsequently denoted as ‘regular’ and ‘irregular’.

**Table 1:** Motion characteristics: maximum and mean peak-to-peak amplitudes, mean breathing cycle lengths.

Case		Variability	max. Amplitude (mm)			ø Amplitude (mm)			ø Period (s)
			SI	AP	LR	SI	AP	LR	
1	a	none (sine)	20	10	0	20	10	0	3.1
	b		20	10	0	20	10	0	4.5
	c		20	10	0	20	10	0	5.8
	d		20	0	0	20	0	0	4.5
	e		20	10	10	20	10	10	4.5
2	a	regular	16.3	11.8	N/A	12.8 ± 3.3	9.4 ± 3.3	N/A	4.8 ± 0.8
	b	irregular	22.5	23.9	N/A	13.0 ± 5.2	13.6 ± 5.5	N/A	4.4 ± 1.0

SI: superior-inferior; AP: anterior-posterior; LR: left-right.

With the tumor insert of the lung phantom defining an 2×2×2 cm clinical target volume (CTV), the internal target volume (ITV) being chosen sufficiently large to compensate for simulated motion patterns, and the lungs surrounding the ITV as organ at risk, dual-arc VMAT (RapidArc, Varian Medical Systems, USA) plans with standard MLC modulation were created using Eclipse 13 (Varian Medical Systems). Taking into account an angular dependency of the Octavius 1000 SRS detector array as reported in [28] (larger differences between measured dose and the dose calculated by the treatment planning system for dose delivery to the rear of the detector), we restricted the utilized gantry angle interval to 285° to 75° and vice versa, cf. Fig 1. Thus, beam incidence is always on the front of the detector (the angle perpendicular to the detector front plane is 360°/0°). ITV dose coverage was optimized based on the average CT corresponding to a 10-phase 4D CT of the moving phantom [motion pattern 1b of Table 1; CT scanner: Siemens Definition AS+ (Siemens Healthcare, Germany) with Real-Time Position Management system (Varian Medical Systems)]. For 6 Gy prescribed dose, a nominal 600 MU/min dose rate and an energy of 6 MV, the ‘beam on’ time per arc was 50 s, corresponding to a mean arc velocity of 3°/s.

#### Experiments I: dose measurements

The treatment plans were delivered without (static measurement) and with phantom motion (dynamic measurement) by a TrueBeam linear accelerator (Varian Medical Systems, USA). The delivered dose was separately measured for the individual arcs, and two measurement series were acquired at different days for each motion scenario to estimate related dose measurement uncertainties. The motion phantom position for static measurements corresponded to mid-respiration states of the individual motion scenarios. This position also represented the starting position and breathing phase for the dynamic measurements (synchronized with ‘beam on’ signal of the treatment unit).

#### Experiments II: 4D VMAT dose simulation

As the motion phantom was limited to rigid movements and to be able to separately analyze the impact of parameters P2 and P3, we explicitly refrained from using image-based obtained motion trajectories *φ*_*i*,*j*,*t*_. Instead, the trajectories programmed to the phantom and used throughout the measurements were applied as *φ*_*i*,*j*,*t*_ in Eq (4). With a resolution of 0.04 s, uncertainties arising from temporal motion trajectory discretization were assumed to be negligible. To further illustrate the influence of inaccuracies regarding the synchronization of the breathing phases at motion phantom measurement and simulation beginning (P1), 4D dose simulation has been repeated with systematically varied starting phase. This part of the experiments was also intended to demonstrate potential advantages of a simulation-based approach in comparison to measurement studies.

With the motion trajectories being assumed to agree between dose measurements and simulation, the influence of the temporal discretization of the dose delivery process (P2) was studied by varying the angle interval size Δ*α*. Four discretization levels were applied: 2.3° (corresponds to 65 segments per arc), 5° (30 segments), 10° (15 segments), and 150° (1 segment). The corresponding angle interval dose segments *D*_*α*_ were determined using the arc splitting option implemented in Eclipse for verification purposes. The smallest possible angle interval size in Eclipse was 2.3°, which explains the aforementioned choice for the finest discretization level. The last discretization choice actually means that the planned arc is not split into segments. This, in turn, represents the situation that effects of dynamically changing beam parameters are not accounted for during 4D dose simulation; the geometry is moved inside the originally planned ‘dose cloud’ and interplay effects are neglected. The hypothesis was that, if interplay effects influenced the measurements, the agreement between measurement and simulation should increase with finer discretization.

For studying the influence of differences with respect to calculation of the static dose distributions *D*_*i*,*j*,*α*_, 4D dose simulation was repeated with the arc segment dose distributions being computed based on the intensity distribution of the average CT, based on a single phase CT of the acquired 4D CTs, and exploiting the entire set of breathing phases represented by the 4D CTs. In the latter case, a dose distribution *D*_*i*,*j*,*α*_ was assumed to correspond to the dose distribution computed by means of the CT with the breathing phase closest to the actual phase represented by *φ*_*i*,*j*,*t*_. In addition, the outputs of the dose calculation algorithms applied to lung SBRT in our facility–the analytical anisotropic algorithm (AAA) and Acuros XB–were exemplarily compared.

#### Evaluation and comparison of measured and simulated dose distributions

Measured and simulated dose distributions were primarily compared by 2D-*γ*-evaluation [29], with *γ*-value maps and *γ*-passing rates analyzed for the CTV, ITV and the entire measurement area determined by the flat panel detector [30]. The coronal slice of the simulated 3D dose distributions that was selected for comparison to the measured dose distributions corresponded to the slice of the planning average CT that, in terms of visual inspection, most closely corresponded to the central plane of the detector (cf. Fig 1). During plan generation, the center of the detector plane visible in the coronal CT slice was further marked by a reference point. Centered in this point, a 2D-region of interest (ROI) of similar size as the detector area was extracted from the coronal dose slice, resampled to the spatial resolution of the measurement dose distribution, and remaining small spatial shifts caused by imperfect phantom setup were manually corrected. In agreement with standard QA parameters, a local *γ*-criterion of 3%/3 mm was applied and only pixels with dose values ≥ 20% of the maximum dose value were considered. Software packages used for evaluation were Verisoft 6.0 (PTW Freiburg, Germany) and Matlab R2015a (MathWorks, USA). All measured and simulated 2D dose ROIs underlying subsequent result tables and figures are provided as supporting information (S1 File) to allow interested readers to reproduce the data.

## Results

**Fig 3:**
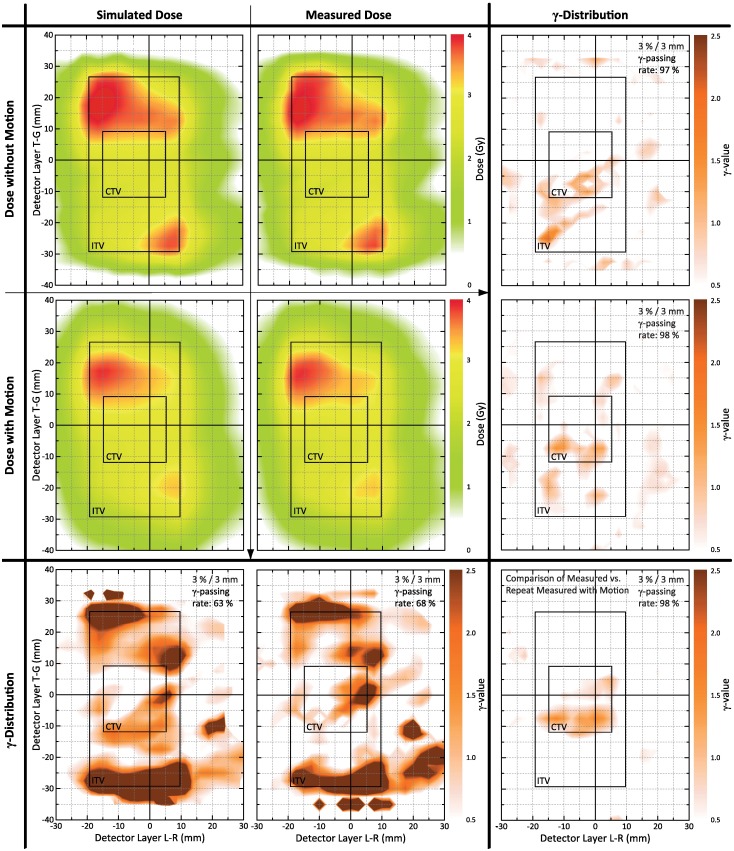
Study design and evaluation strategy. Illustration of performed experiments for the SI-only sinusoidal motion with 4.5 s period (i. e. case 1d); for details see text. Left column: planned dose distribution (top), simulated motion-affected dose (middle; arc discretization of 2.3°), *γ*-map for comparison of the two (bottom). Middle column: measured static dose (top), measured dynamic dose (middle), *γ*-comparison (bottom). Right column: *γ*-comparison of planned and measured static dose (top), *γ*-comparison of simulated motion-affected and corresponding measured dose (middle), *γ*-comparison of repeat dynamic measurements (bottom).

Fig 3 illustrates the concept underlying our study design and evaluation strategy: Focusing on a single VMAT arc of a dual-arc treatment plan, the left column of the figure shows the planned dose distribution (‘simulated dose without motion’; top), the accumulated dose representing the simulated motion effects (‘simulated dose with motion’; middle), and the *γ*-map demonstrating differences between the two distributions, i. e. the simulated motion effects (*γ*-criterion 3%/3 mm; bottom). The middle column represents the same information for the measurements (from top to bottom: static measurement, measurement with motion, *γ*-map for comparison of static and moved measurement).

Focussing on the question of the appropriateness of simulation-only based 4D VMAT QA and therefore being primarily interested in an assessment of the 4D VMAT dose simulation accuracy and parameters affecting it, the motion effects represented in the left and middle *γ*-maps were only of secondary interest; instead, it was the agreement of the two *γ*-maps as well as the similarity of underlying simulated and measured motion affected dose distributions that was to appraise. The motivation for choosing the given arc and its inhomogeneous dose distribution within the ITV as a showcase was in line with this argument: the inhomogeneous dose distribution simplified visual assessment of motion effects and respective differences in comparison to homogeneous high ITV doses that would result for single arc treatment planning.

Direct comparison of simulated and measured motion-affected dose distributions by means of the corresponding *γ*-map is finally illustrated in the middle of the right column. For ideal agreement, the *γ*-map would correspond to an 100% *γ*-passing rate. This ideal scenario is, however, not realistic due to unavoidable uncertainties influencing the comparison. The figure represents two such sources. In the right top corner, the planned dose and the static measurement were compared. The *γ*-map corresponds to a total *γ*-passing rate of 97%; related uncertainties, of course, also affect a comparison of dynamic simulated and measured doses. In addition, the right bottom corner provides differences between repeat dynamic measurements; the *γ*-passing rate was 98%. These reference values have to be taken into account for discussion of *γ*-passing rates between measured and simulated motion-affected dose distributions. Nevertheless, for the shown case, the *γ*-passing rate between dynamic measurement and motion-affected simulation was still 98%.

### Influence of VMAT arc discretization on 4D VMAT dose simulation accuracy

**Fig 4:**
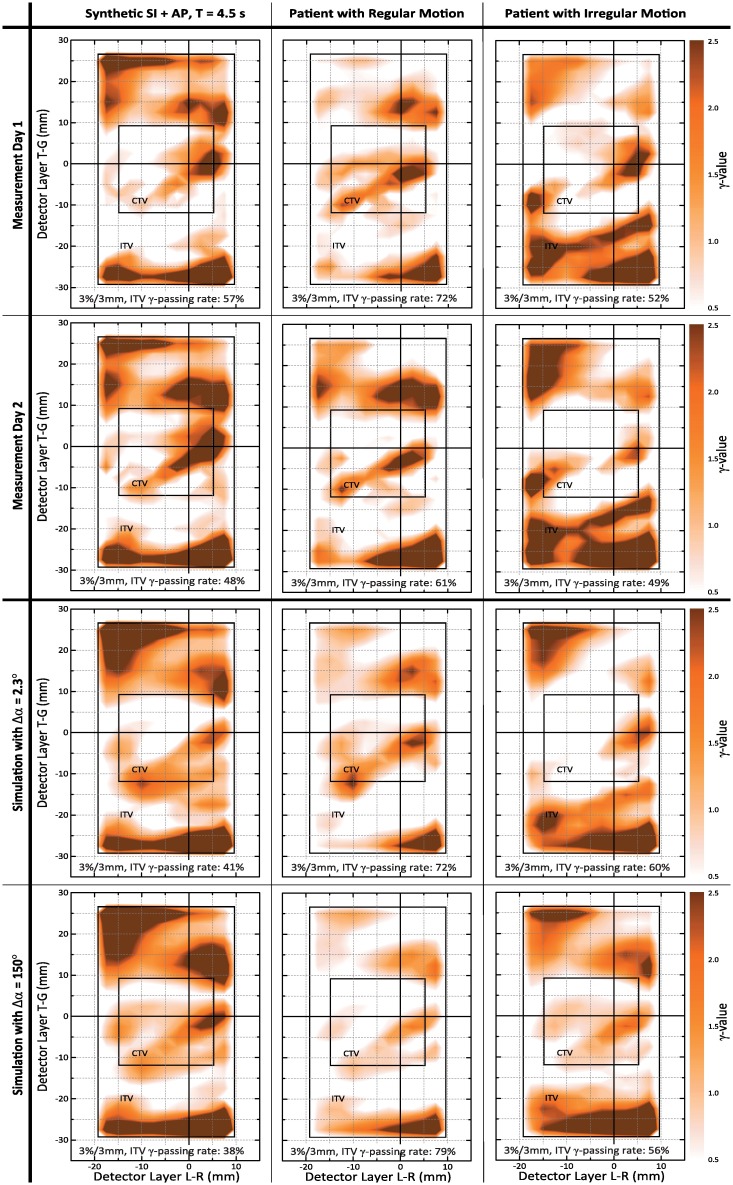
Influence of arc discretization. Illustration of the influence of arc discretization on simulated motion effects. 3^rd^ row: *γ*-comparison to planned dose for finest possible arc discretization; 4^th^ row: no discretization. Results have to be compared to *γ*-maps between static and motion-affected measurements in 1^st^ and 2^nd^ row. Differences between the simulation *γ*-maps and the measurement *γ*-maps should be as small as possible.

The influence of the degree of temporal discretization of the technical dose delivery process on the 4D VMAT dose simulation results is illustrated in Fig 4, again using the first arc of the respective dual-arc VMAT plans. The figure shows *γ*-maps obtained by comparison of static and dynamic measurements (first two rows) and *γ*-maps for comparison of the statically planned and simulated motion-affected dose distributions (last two rows); the columns represent different motion scenarios. The *γ*-maps therefore represent measured and simulated motion effects. Consequently, a simulation is superior to another when its *γ*-map more closely resembles the *γ*-map of the measurement.

For the regular motion patterns, the *γ*-maps obtained for both Δ*α* = 2.3° and Δ*α* = 150° simulations appear to well agree with the *γ*-maps for the measurements–especially, when considering between-measurement differences (comparison of rows 1 and 2). Deviations between measurement and simulation increased, however, for the irregular motion pattern (see right column), with the agreement between the Δ*α* = 150° *γ*-map and the measurements being noticeably smaller than for Δ*α* = 2.3°. This already indicated that taking into account the interplay between the dynamic dose delivery process and target structure motion on a fine-scale temporal resolution allows for a more reliable computational simulation of the VMAT-delivered motion-affected dose.

The aforementioned impression was further supported by the quantitative evaluation of the *γ*-maps summarized in Tables 2–5. The visual agreement between measurement- and simulation-based *γ*-maps for regular motion, independent of the degree of arc discretization, is mirrored by high *γ*-passing rates when directly comparing the simulated motion-affected dose distributions and the dynamic measurements; cf. Table 2. In turn, the visual differences for scenario 2b (3^rd^ column of Fig 4) translated into a drop of the *γ*-passing rate from 97% (Δ*α* = 2.3°) to 82% (Δ*α* = 150°).

**Table 2:** Total *γ*-passing rates for comparison of dynamic dose measurements of day 1 (D_dyn,day1_) and dynamic day 2 dose measurements and simulated 4D dose distributions (D_dyn,⋅_) with respect to the impact of the arc discretization on the simulation accuracy.

	D_dyn,day1_ vs. D_dyn,⋅_ [%]							$\bar{\gamma }$ [%]
	1a	1b	1c	1d	1e	2a	2b	
Day 2 measurement	99	98	98	98	99	99	100	**99 ± 1**
4D VMAT simulation: 65 seg. (2.3°)	99	98	98	98	98	100	97	**98 ± 1**
4D VMAT simulation: 30 seg. (5°)	98	97	96	97	96	99	94	**97 ± 1**
4D VMAT simulation: 15 seg. (10°)	99	94	91	94	95	96	91	**94 ± 3**
4D VMAT simulation: 1 seg. (150°)	99	96	93	95	95	97	82	**94 ± 5**

$\bar{\gamma }$ represents the mean gamma passing rates, averaged over all motion scenarios. The closer the numbers to 100%, the better. Ideally, *γ*-passing rates between day 1 measurements and 4D VMAT simulations are in the same order than those for comparison of day 1 and day 2 measurements.

**Table 3:** Total *γ*-passing rates for comparison of static dose measurements to dynamic measurements (lines ‘Day 1’ and ‘Day 2’) and *γ*-passing rates for comparison of the statically planned dose and the dose distributions containing simulated motion effects.

	D_sta,⋅_ vs. D_dyn,⋅_ [%]							${\bar{\Delta }}_{\gamma }$ [%]
	1a	1b	1c	1d	1e	2a	2b	
Day 1 measurement	65	67	64	68	62	86	68	**–**
Day 2 measurement	59	62	61	62	60	79	68	**4.1 ± 2.4**
4D VMAT simulation: 65 seg. (2.3°)	61	60	57	63	60	86	69	**3.7 ± 2.6**
4D VMAT simulation: 30 seg. (5°)	61	60	56	62	61	85	68	**3.9 ± 3.0**
4D VMAT simulation: 15 seg. (10°)	61	58	54	59	59	87	67	**5.3 ± 3.7**
4D VMAT simulation: 1 seg. (150°)	60	59	60	60	60	89	74	**5.1 ± 2.2**

Cf. Table 2 for symbols and indices. Comparing static to dynamic measurements / simulations, the simulations should now (different to Table 2) resemble the numbers of the measurements as closely as possible. Consequently, the absolute difference ${\bar{\Delta }}_{\gamma }$ between the *γ*-passing rates obtained by the simulations and the *γ*-passing rates of the day 1 measurements should be (averaged over all motion scenarios) as low as possible and in the same order as the differences between day 1 and day 2 measurements.

**Table 4:** CTV *γ*-passing rates for comparison of static dose distributions and dynamic dose measurements/simulations.

	CTV: D_sta,⋅_ vs. D_dyn,⋅_ [%]							${\bar{\Delta }}_{\gamma }$ [%]
	1a	1b	1c	1d	1e	2a	2b	
Day 1 measurement	45	73	59	73	73	59	80	**–**
Day 2	32	59	41	55	71	71	82	**11.3 ± 6.3**
4D VMAT simulation: 65 seg. (2.3°)	66	68	57	68	82	71	93	**9.6 ± 6.0**
4D VMAT simulation: 30 seg. (5°)	70	66	45	64	77	68	86	**10.6 ± 6.6**
4D VMAT simulation: 15 seg. (10°)	63	54	27	41	68	88	79	**19.4 ± 11.7**
4D VMAT simulation: 1 seg. (150°)	59	57	59	48	86	93	82	**14.9 ± 11.1**

Cf. Table 2 for symbols and indices. Similar to Table 3, a simulation result is considered superior to another if its *γ*-passing rate values are closer to corresponding measurement values.

**Table 5:** ITV *γ*-passing rates for comparison of static dose distributions and dynamic dose measurements/simulations.

	ITV: D_sta,⋅_ vs. D_dyn,⋅_ [%]							${\bar{\Delta }}_{\gamma }$ [%]
	1a	1b	1c	1d	1e	2a	2b	
Day 1 measurement	48	57	47	58	50	72	52	**–**
Day 2 measurement	38	48	41	46	48	61	49	**7.6 ± 3.7**
4D VMAT simulation: 65 seg. (2.3°)	43	41	33	42	44	72	60	**9.3 ± 5.7**
4D VMAT simulation: 30 seg. (5°)	43	41	29	40	46	71	56	**9.4 ± 7.0**
4D VMAT simulation: 15 seg. (10°)	42	35	26	32	41	76	53	**12.7 ± 9.3**
4D VMAT simulation: 1 seg. (150°)	39	38	39	35	48	79	56	**10.3 ± 7.2**

Cf. Table 2 for symbols and indices and Table 4 for further explanations.

A similar tendency could be observed when directly studying the *γ*-passing rates obtained by comparison of, on the one hand, static and dynamic measurements, and, on the other hand, *γ*-passing rates for comparison of statically planned and simulated motion-affected dose distributions (i. e. *γ*-passing rates corresponding to, for instance, the *γ*-maps of Fig 4). As these *γ*-passing rates directly quantify measured and simulated motion effects, the simulation values should be as close as possible to the measurement values. In turn, related differences were assumed to indicate simulation uncertainties. Table 3 already reflects that the average differences of simulation to measurement *γ*-rates are smallest for the finest discretization level Δ*α* = 2.3°. The differences become, however, more pronounced when switching from total to structure-based *γ*-rates (i. e. when focusing on higher dose areas) shown in Tables 4 and 5. In this case, Δ*α* = 2.3° not only resulted in lowest ${\bar{\Delta }}_{\gamma }$ values but also (more or less on a par with Δ*α* = 5°) *γ*-passing rates differences to the day 1 measurement that were in the order of the day 2-to-day 1 measurement differences for, e. g., the CTV.

Observations for the other arcs of the treatment plans were similar.

### Influence of starting phase/synchronization uncertainties

**Fig 5:**
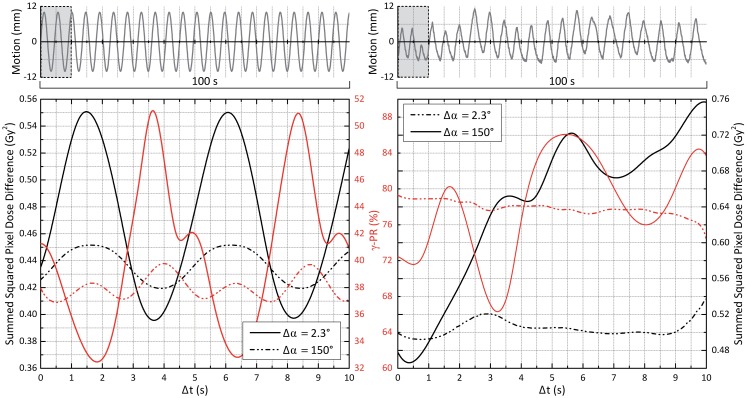
Starting phase influence. Influence of breathing phase at dose delivery beginning. Left, top: In accordance with the measurements, all previous results were computed with the simulations starting at the breathing phase at *t* = 0 s of the curve (here: case 1b). Now, this starting phase was systematically varied by adding offsets Δt∈[0s;10s]. Left, bottom: The ITV *γ*-passing rates for comparison of planned static and motion-affected simulated dose distributions are shown as red lines (solid lines: Δ*α* = 2.3°; dashed: Δ*α* = 150°); the black lines visualize the dependence of the difference between dynamic measurement and simulated motion-affected dose on the starting phase. Right: similar information but for the regular real tumor trajectory (case 2a).

So far, perfect agreement between the initial breathing phase of the motion phantom at dose delivery beginning and the breathing phase applied to *t* = 0 s of the simulations was assumed. As signal latencies potentially led to phase shifts between measurement and simulation, in the next step, the breathing phase at *t* = 0 s of the simulations was systematically varied. The effect on the ITV *γ*-passing rates between statically planned and simulated motion-affected dose on the one hand and absolute pixel-wise squared dose differences (SDD) between dynamic measurement and simulation on the other hand are shown in Fig 5 for synthetic (case 1b) and real (case 2a) motion scenarios. For both Δ*α* = 2.3° and Δ*α* = 150°, it can be seen that the SDD values were close to minimum for Δ*t* = 0 s, with the actual SDD minima, however, being slightly shifted in time (in negative direction for case 1b, in positive direction for case 2a).

In addition to the illustration of uncertainties due to mismatches of measurement and simulation starting phases, the figure also demonstrates the dependence of predicted VMAT interplay effects on the assumed starting phase (in terms of *γ*-passing rates between static planned and simulated motion-affected dose distributions) by varying Δ*t* over a longer period (here: 10 s). It becomes obvious that the Δ*α* = 2.3° simulations (beforehand proven to be the most accurate ones) predicted a substantial impact of the starting phase on the *γ*-passing rate and amount of interplay effects, respectively–whereas such details were not reflected by the Δ*α* = 150° simulations.

### Static dose calculation uncertainties

In agreement with Ref. [23, 31], uncertainties related to static dose calculation approaches proved subordinate to the temporal discretization and the assumed breathing phase at the beginning of dose delivery. 3%/3 mm *γ*-comparison of simulated motion-affected dose distributions with the doses *D*_*i*,*j*,*α*_ computed based on the closest neighbored phase CT, a fixed single phase CT or the average CT resulted in total *γ*-passing rates between 98% and 100% for all motion scenarios. Uncertainties due to AAA or Acuros XB dose calculation were in the order of ±3%.

## Discussion and conclusions

Being placed in the context of the current discussion of appropriate VMAT 4D quality assurance approaches, the present study aimed at assessing 4D VMAT dose simulation accuracy and associated influencing parameters by comparing simulated motion-affected dose distributions to corresponding ground truth dynamic measurements–and so study potential limitations of computational simulation-only 4D VMAT QA to assess appropriateness of a VMAT treatment plan when taking into account patient-specific breathing and motion characteristics.

We identified and illustrated the temporal discretization of the dose delivery process as major technical factor and the breathing phase at dose delivery beginning as most relevant physiology/breathing curve-related parameter. Applying the highest VMAT arc discretization of 2.3° achievable by the treatment planning system, high total *γ*-passing rates of on average 98% between simulations and dynamic measurements [cf. Table 2] rise, from our perspective, the question whether measurement-based assessment of VMAT motion effects still remains necessary or if it can be replaced by 4D VMAT dose simulations–with our opinion being the latter.

As 4D VMAT dose simulation accuracy has been shown to decrease for arc discretization levels larger than 2.3°, remaining small differences to the measurements may be in parts due to the mentioned software limitations (i. e., < 2.3° discretization not possible). However, uncertainties such as existence of a potential shift between breathing phases at dose measurement and simulation beginning, uncertainties of the applied dose calculation algorithm (although illustrated to be small), and, e. g., the angular and dose rate dependence of the detector array (although also reported to be small for the gantry angles and dose rates exploited in our study, cf. [28] for respective details) are likely to also affect the numbers. This superposition of uncertainties motivates further detailed studies.

In terms of limitations of our study, we would again emphasize that we explicitly refrained from analyzing uncertainties introduced by registration errors associated to, e. g., motion estimation in clinical 4D CT data. This has been in parts due to the motion phantom design (only rigidly moving phantom); we, however, also believe that uncertainty estimation and quantification of deformable image registration represents an issue that has yet not been solved in its entirety. We consider this topic to be beyond the scope of this study but to represent an important aspect of our future work.

As a consequence of the aforementioned limitation, the impact of potential breathing-induced deformation on, e. g., the interplay effect, target coverage and the numbers presented in Tables 3 to 5 remains unclear. This aspect could be of interest in terms of future work as (although not being the primary focus of our QA-oriented study) our results indeed demonstrate interplay effects and clinically relevant low CTV *γ*-passing rates for at least our single-arc scenarios; and even for the full dual-arc plans and simulations, low CTV *γ*-passing rates < 60% could be observed for irregular and longer breathing period motion, despite the well-reported averaging effect for multiple arcs [10, 23]. However, these results are obtained using only rigid motion patterns. In addition, the interplay effects are shown to depend on the breathing phase at dose delivery beginning, and we would also like to note that only a standard dose rate of 600 MU/min was used in the current study. Higher dose rates, e. g. provided by flattening filter-free (FFF) dose delivery, are likely to result in a further increased risk of VMAT interplay effects [32]. A detailed discussion of such aspects and related questions (How to account for potentially different breathing phases at the beginning of VMAT dose delivery during 4D QA? How to account for breathing variability during 4D QA? How to stabilize 4D VMAT treatment planning in terms of a robust target dose coverage even in the presence of motion variability?) would again be beyond the scope of this paper but motivates additional studies.

## Supporting information

S1 File File containing dose distributions underlying the manuscript figures and numbers. The zip-file contains the simulated 4D VMAT dose distributions and the the dose measurements (serving as ground truth data) that are underlying the figures and numbers presented in the manuscript. For further details see the Readme.md file contained in the zip-file.
(ZIP)
